# Odor Learning and Its Experience-Dependent Modulation in the South American Native Bumblebee *Bombus atratus* (Hymenoptera: Apidae)

**DOI:** 10.3389/fpsyg.2018.00603

**Published:** 2018-04-27

**Authors:** Florencia Palottini, María C. Estravis Barcala, Walter M. Farina

**Affiliations:** ^1^Laboratorio de Insectos Sociales, Departamento de Biodiversidad y Biología Experimental, Facultad de Ciencias Exactas y Naturales, Universidad de Buenos Aires, Buenos Aires, Argentina; ^2^Instituto de Fisiología, Biología Molecular y Neurociencias (IFIBYNE), CONICET – Universidad de Buenos Aires, Buenos Aires, Argentina

**Keywords:** bumblebee, associative learning, latent inhibition, odor pre-exposure, *Bombus atratus*

## Abstract

Learning about olfactory stimuli is essential in bumblebees’ life since it is involved in orientation, recognition of nest sites, foraging efficiency and food yield for the colony as a whole. To evaluate associative learning abilities in bees under controlled environmental conditions, the proboscis extension response (PER) assay is a well-established method used in honey bees, stingless bees and successfully adapted to bumblebees of the genus *Bombus*. However, studies on the learning capacity of *Bombus atratus* (Hymenoptera: Apidae), one of the most abundant native species in South America, are non-existent. In this study, we examined the cognitive abilities of worker bees of this species, carrying out an olfactory PER conditioning experiment. Bumblebees were able to learn a pure odor when it was presented in paired association with sugared reward, but not when odor and reward were presented in an unpaired manner. Furthermore, if the bees were preexposed to the conditioned odor, the results differed depending on the presence of the scent either as a volatile in the rearing environment or diluted in the food. A decrement in learning performance results from the non-reinforced pre-exposure to the to-be-conditioned odor, showing a latent inhibition phenomenon. However, if the conditioned odor has been previously offered diluted in sugared reward, the food odor acts as a stimulus that improves the learning performance during PER conditioning. The native bumblebee *B. atratus* is thus a new hymenopteran species capable of being trained under controlled experimental conditions. Since it is an insect increasingly reared for pollination service, this knowledge could be useful in its management in crops.

## Introduction

Bumblebees of the genus *Bombus* (Hymenoptera: Apidae) are social insects with an annual life cycle which play an important role as pollinators in natural and agricultural ecosystems. For this reason, presently, their colonies are commercialized to improve the production of diverse crops (Heinrich, 2004). However, the worldwide trade in bumblebee colonies for crop pollination, in particular of *B. terrestris*, has elicited special concern about the potential for invasion by non-native bumblebees and their impacts on native pollinator species (Morales et al., 2013).

*Bombus atratus* Franklin is present in almost all South American countries, except for northern Brazil, Guyana, and Chile (Abrahamovich et al., 2001). It is the most widely distributed and most abundant bumblebee species in Argentina, with great climatic and altitudinal tolerance (Abrahamovich et al., 2001). Because of a clear evidence about their efficiency to pollinate diverse crops grown under cover as tomatoes, eggplants, sweet peppers, blueberries and kiwifruits; colonies of this native species, as others species of the same genus, are commercialized to improve the plant production in pollination services (Aldana et al., 2007; Basualdo et al., 2013; Godoy et al., 2013; Alvarez et al., 2014; Riano et al., 2015).

Learning about olfactory stimuli is essential in bumblebees’ life. In particular, in an appetitive context, when collecting at a flower, bees establish an associative memory between a floral scent and the nectar reward, setting out a contingency between the Conditioned Stimulus (CS, floral odor) and the Unconditioned Stimulus (US, nectar). In this way, associative learning represents the basis for efficient foraging behavior in bees, because it allows them to relocate specific food sources and efficiently collect pollen and nectar from different species of flowers. Indeed, bumblebee’s foragers are able to learn the quality (in terms of nectar sugar concentration) of the flowers they visit and subsequently tend to specialize on the more profitable species (Cnaani et al., 2006). Furthermore, bumblebees possess the ability to learn and use memories to discriminate flowers on the basis of diverse floral properties, including morphology, color, scent and nectar quality (Dukas and Real, 1993; Chittka et al., 1997; Gumbert, 2000; Spaethe et al., 2007; Leonard et al., 2011).

Examples that bumblebees modify their performance during the search for food outside the nest if they experienced scented nourishment that circulated inside the colony have been reported previously (Dornhaus and Chittka, 1999; Molet et al., 2009). However, the nature of the behavioral mechanisms involved in the information transfer process is unknown. The exposure to a neutral stimulus paired or not with the unconditioned one before the training process could affect differently the behavioral response toward the stimulus to be conditioned (Mackintosh, 1994). If the experimental subject was previously exposed to a CS without pairing with the US and the acquisition of an association is delayed, this phenomenon is defined as latent inhibition, LI (Lubow and Moore, 1959; Lubow, 1973). Contrarily, previous experiences of the CS paired with the US might act as a stimulus that improves associative learning (Mackintosh, 1994). Non-associative processes could also occur, such as the case of sensory pseudoconditioning, where an increase in the response is observed just by the repeated presentation of reinforcement, or sensory priming, in which a preexposed sensory stimulus such as an odor influences a response to a subsequent stimulus of the same sensory modality (Bouton and Moody, 2004). Thus, the assessment by using a standardized learning protocol with individuals of known experience is a way to determine if the mechanisms involved are of sensory or cognitive nature.

The proboscis extension reflex (PER) is part of the behavior to search for food inside the nectaries and allows worker bees to draw up nectar and pollen from flowers. Under controlled environmental conditions, the PER is a well-established method used in honey bees (Takeda, 1961; Bitterman et al., 1983), stingless bees (Mc Cabe et al., 2007; Mc Cabe and Farina, 2009, 2010) and some species of the genus *Bombus* (Laloi et al., 1999; Riveros and Gronenberg, 2009; Toda et al., 2009; Sommerlandt et al., 2014; Lichtenstein et al., 2015), that allows researchers to evaluate associative learning abilities. However, until now, the learning capacity of the native South American *B. atratus* species is unknown.

Bearing this in mind, the present research aimed to examine the cognitive capacity of *B. atratus* worker bumblebees, performing an olfactory classical PER conditioning procedure. First, we evaluated the bumblebees’ ability to associate an odorant cue with reinforcement. Furthermore, pre-exposure protocols were applied to analyze the influence of previous experiences in the learning performances. On the one hand, to evaluate the presence of a latent inhibition phenomenon, we performed an odor pre-exposure in the environment. Finally, in another experiment, we evaluated the effect of the prestimulation with a scented sugar solution with the odor to be used as CS in the classical conditioning.

This is the first report about odor learning abilities in the South American native bumblebee *B. atratus.*

## Materials and Methods

### Study Site, Animals, and Odorant Cues

Eleven bumblebee colonies (*B. atratus* Franklin) were provided by Biobest Argentina S.A. (Burzaco, Province of Buenos Aires, Argentina) and maintained in the laboratory at the Experimental Field of the University of Buenos Aires, Argentina (34° 32′S, 58° 26′W). All experiments were carried out during the summer-autumn season of 2017 and 2018. The colonies were housed in their original commercial boxes (27 cm × 24 cm × 20 cm). The boxes were kept in the laboratory under natural daylight conditions filtered through window glass and fed *ad libitum* with a sugar solution provided by the supplier and honey bee-collected pollen.

A pool of seven colonies was used to carry out Experiment 1, while six colonies were allocated to Experiments 2 and 3. To exclude colony effects, individuals of the assigned colonies contributed to the data of the experimental and the corresponding control series within each experiment.

Pure odors commonly presented in the floral fragrances (Knudsen and Tollsten, 1993; Raguso and Pichersky, 1999), such as the case of linalool (LIO), phenylacetaldehyde (PHE) and nonanal (NONA; Sigma-Aldrich, Steinheim, Germany), were used during the experiments.

### Bees’ Capture and Harnessing

Colonies were anesthetized with carbon dioxide and individual workers of unknown age and various sizes (intertegula span between 2.4 and 4.44 mm) were randomly captured and confined in wooden cages (10 cm × 10 cm × 10 cm) in groups of 10–15 individuals, to reduce the stress level and increase the survival rate (personal observation during bees manipulation). Bees were fed *ad libitum* with 1.8 M unscented sucrose solution and kept in darkness in an incubator for 2 h at 25°C and 75% relative humidity.

**FIGURE 1:**
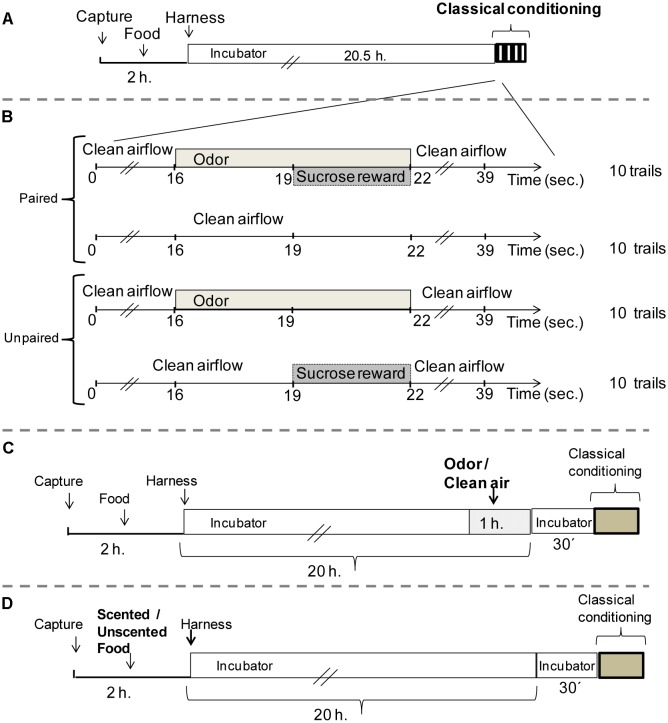
Protocols to examine associative learning in *Bombus atratus* worker bumblebees. **(A)** Protocol prior to perform an associative classical conditioning proboscis extension in bumblebees (Experiment 1). **(B)** Detail of paired and unpaired training (Experiment 1). **(C)** Volatile pre-exposure (Experiment 2), bumblebees were preexposed in the environment to 60 μl of pure odor inside a plastic box during 1 h or not preexposed (control). **(D)** Prestimulation with a scented food (Experiment 3), bumblebees were fed with 20–40 μl of the scented food (50 μl of pure odorant/liter of 1.8M sucrose solution) or with unscented food (1.8M sucrose solution).

Experimental bees were then anesthetized and harnessed in metal tubes so that only the antennae and mouthparts could freely move. Bees were fed with 1.8 M unscented sucrose solution and kept in the incubator for 20.5 h under the same conditions previously described, prior to olfactory conditioning (**Figure 1A**). Once the time has passed, a restrained bumblebee was placed individually in front of the device used for application of the odorant during the conditioning protocol.

### Behavioral Assays

#### Proboscis Extension Response Protocol

Bumblebees underwent a classical conditioning protocol adapted from the proboscis extension response (PER) paradigm, which is well established in honey bee olfactory learning procedure (Takeda, 1961; Bitterman et al., 1983). To assay the PER, a device that delivered a continuous airflow (50 ml/s) was used for the application of the odorant. Four microliters of pure odorant impregnated on 30 × 3 mm filter paper inside a syringe were delivered through a secondary air-stream (6.25 ml/s) to the head of the bee. A fan extracted the released odors to avoid contamination (Fernández et al., 2009). Each learning trial lasted 39 s. Before odor presentation, bees rested for 15 s in the airflow for familiarization as well as for testing the bees’ response toward the mechanical stimulus. For the training procedure of the classical conditioning, we presented the CS for 6 s. Reinforcement (1.8 M sucrose solution) was presented on the proboscis (mouthparts) and occurred for 3 s, 3 s after the onset of the CS. Memory retention tests were performed 10–15 min after the last conditioning trial and consisted of the presentation of the CS and of a novel odor (NO), both without reinforcement. We considered the PER during the first 3 s of the presentation of the test odor. The order of presentations of the two odors was chosen at random prior to the onset of the test to avoid possible sequential effects. Thus, half of the subjects were tested with the CS first and the NO second, while the other half, with the reversed sequence. Only bees that did not respond to the mechanical airflow stimulus were used.

#### Experiment 1: Olfactory Classical Conditioning

As an initial approach to study if native bumblebees have the ability to associate an odorant cue with reinforcement, we performed an odor classical conditioning with a pure odor as CS, LIO. A second pure odor was used as novel odor during the testing phase, nonanal (NONA). Bumblebees underwent 10 training trials of paired CS-US presentations. In addition to the paired group, for which the presentation of the CS (LIO) was paired with the US, another group received unpaired presentations of the CS and of the US in a pseudo-randomized sequence, as an explicitly unpaired control group (Matsumoto et al., 2012). Both groups underwent a total of 20 trials. The paired group was subject to 10 training trials of paired CS-US presentations and 10 blank trials in between, in which each bee was placed in the setup without any stimulation for 39 s. Thus, both groups had exactly the same sensory experience (10 CS and 10 US presentations) with an average ITI of 10 min (**Figure 1B**). Retention tests were performed 10 min after the last training trial. Those bees that extended their proboscis in the first trial during the odor presentation (innate response) were excluded and they did not finalize the training protocol.

To determine whether increases in conditioned responses in the absolute conditioning were a consequence of associative learning and did not depend on the odor identity, a different pure odor, PHE, was used as CS in a second series of this experiment following the same protocol described above. In this series, retention tests were performed 15 min after the last conditioning trial and consisted of presentations of the CS and of a novel odor (NONA), both without US.

#### Olfactory Stimulation Before Conditioning

To study the influence of previous odorant experiences in the learning performance at the PER setup in *B. atratus* bumblebees, harnessed individuals were subjected to volatile pre-exposure in the environment by using the same odor to-be conditioned during the training (in order to evaluate the phenomenon of latent inhibition) (**Figure 1C**) or to a prestimulation with a scented sugar solution (to assess the effect of the odor as preconditioned stimulus) (**Figure 1D**).

#### Experiment 2: Volatile Pre-exposure

To carry out the odor exposure, harnessed bees were moved to another incubator (same conditions of temperature, relative humidity, and darkness). There, bees were placed inside a plastic box (20 cm × 10 cm × 6 cm), where 60 μl of pure odor (LIO) was presented in four filter papers (1.5 cm^2^ evaporation surface) located on the sides of the box. To reduce odor accumulation, an air extractor was connected to the incubator. After the odor exposure (1 h), bees were moved back to the first incubator to prevent odor contamination during the non-exposure period before starting the absolute conditioning (30 min). Another group never exposed to the odor was used as control (**Figure 1C**).

#### Experiment 3: Prestimulation With Scented Food

In this case, individual workers were confined in a plastic queen cage. Herein, bees were fed with 20–40 μl of the scented food offered through Multipette^®^ M4-Repeater^®^M4. Odor solutions were obtained by mixing 50 μl of pure odorant (LIO) per liter of 1.8M sucrose solution. Another group of bees fed with unscented sugar solution was used as a control. Once fed, bees were harnessed as described above and located in the incubator (odorless condition) until the time of the conditioning (**Figure 1D**).

### Statistical Analysis

All statistical tests were performed with R v3.3.3 (R Development Core Team, 2016). The PER was assessed by means of generalized linear mixed-effect models (GLMM) following a binomial error distribution and using the glmer function of the lme4 package (Bates et al., 2015). In the case of training, we considered treatment (a two-level factor corresponding to control or odor; control or preexposed) and trials (a ten-level factor corresponding to 1–10 trials) as fixed effects, with each bee included as a random factor. In the case of test, we considered treatment (a two-level factor corresponding to control or preexposed) and odor (a two-level factor: CS or NO) as fixed effects, with each bee included as a random factor. GLMM were simplified as follows: significance of the different terms was tested starting from the higher-order terms model using anova function to compare between models (Chambers and Hastie, 1992). Non-significant terms (*P* > 0.05) were removed (see Supplementary information). We considered the use of GLMM because these models allow analyzing response variables whose errors are not normally distributed, avoiding the transformation of the response variable or the adoption of non-parametric methods (Crawley, 2013).

## Results

### Experiment 1: Olfactory Classical Conditioning

**FIGURE 2:**
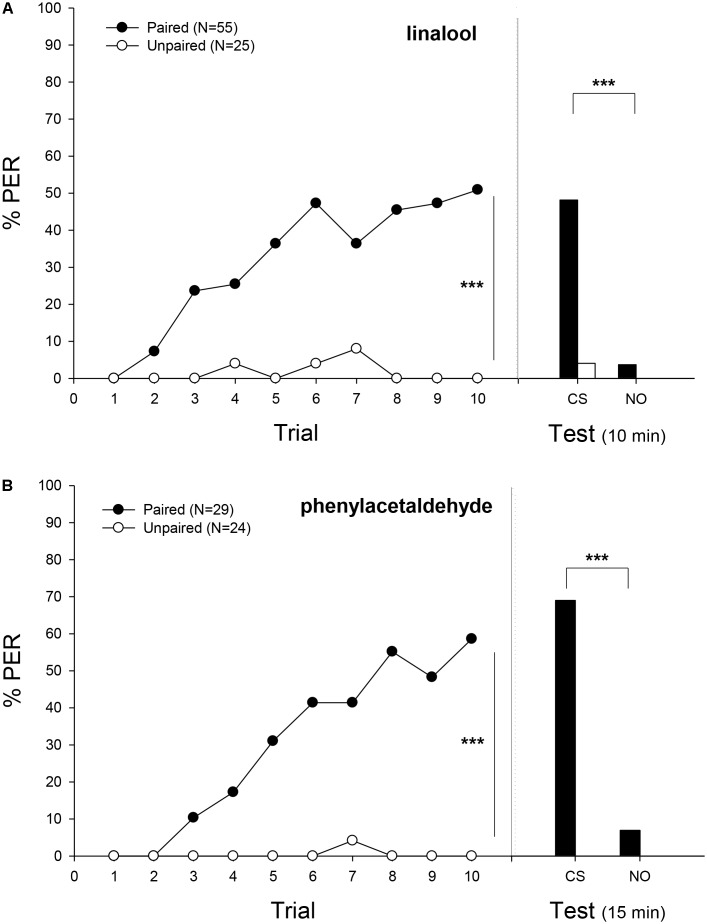
Experiment 1: Olfactory classical conditioning of proboscis extension in bumblebees. Percentage of bees that extended the proboscis as response to the odorant (% PER) during the ten trails in which the conditioned odor was paired (ten reinforced trials, filled circles) or unpaired (ten non-reinforced trials, emptied circles) with the sucrose reward (training, **left panel**) and bees that responded during a testing period 10 or 15 min after training (test, **right panel**). **(A)** Bees were trained with linalool as the conditioned stimulus (CS) and nonanal as novel odor (NO). **(B)** Bees were trained with phenylacetaldehyde as CS and nonanal as NO. In the training phase, the proportion of bumblebees responding to the CS increased with successive conditioning trials, only in the case of paired group (Minimal adequate model: Response ∼ Treatment + Trial + 1| ind.). In the testing phase, bumblebees showed a significantly different response between treatments and between LIO and the novel odor (Minimal adequate model: Response ∼ Treatment + odor + 1| ind.; paired, filled bars; unpaired, emptied bar). In the case of phenylacetaldehyde, bumblebees showed a significantly different response between odors (Minimal adequate model: Response ∼ odor + 1| ind.) Sample sizes are indicated in brackets. Asterisks mean significant differences in the learning performance (*p* < 0.001).

When bees were trained to associate a sucrose reward with LIO as odor stimulus, workers were able to build an association between CS and US after a paired presentation (**Figure 2A**). In the training phase, the proportion of bumblebees responding to the CS increased with successive conditioning trials only in the case of paired group, reaching 51% of conditioned responses at the tenth trial (Minimal adequate model: Response ∼ Treatment + Trial + 1| ind., *p* < 0.001; Supplementary Table S1). In the testing phase, bumblebees showed a significantly different response between treatments (*p* < 0.01) and between LIO and the novel odor (*p* < 0.001; Minimal adequate model: Response ∼ Treatment + odor + 1| ind.; Supplementary Table S1).

When a different odor was used as CS, bumblebees also exhibited associative learning (**Figure 2B**). The acquisition curve for PHE was similar to the one obtained when bees were conditioned to LIO (**Figure 2A**). Bumblebees responded significantly more often to the CS odor in the paired than in the unpaired group (*p* < 0.001), reaching a level of 58% at the tenth trial. The unpaired training group showed negligible levels of response: one bumblebee just responded once at the seventh trial (Minimal adequate model: Response ∼ Treatment + Trial + 1| ind; Supplementary Table S1). In the testing phase, the statistical analysis (GLMM) was only carried out taking into account the paired group because of the lack of response in the unpaired group. Herein, bumblebees presented significantly higher responses to the CS than to the NO (Minimal adequate model: Response ∼ odor + 1| ind, *p* < 0.001; Supplementary Table S1).

Moreover, in order to rule out the possibility that the results of the memory test were not caused by an insensitivity of the bees to nonanal, we performed the conditioning protocol with this odor as CS and LIO as NO (Supplementary Figure S1).

### Olfactory Stimulation Before Conditioning

#### Experiment 2: Volatile Pre-exposure

**FIGURE 3:**
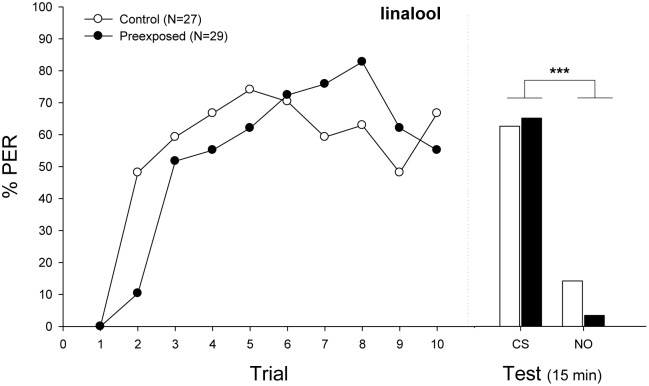
Experiment 2: Effects of volatile pre-exposure in bumblebees classical conditioning. Percentage of bees that extended the proboscis as response to the odorant (% PER) during the ten trails in which the conditioned odor was paired with sucrose reward (training, **left panel**) and bees that responded during a testing period 15 min after training (test, **right panel**). Bumblebees were exposed (filled circles) or not (emptied circles) to the conditioned odor linalool (CS) before olfactory conditioning. In the first trials, preexposed bumblebees exhibited a lower response (Minimal adequate model: Response ∼ Treatment × Trial + 1| ind.). In the testing phase, bees of both groups responded equally well, showing a significantly different response between odors (Minimal adequate model: Response ∼ odor + 1| ind.; preexposed, emptied bars; control, filled bars). Sample sizes are indicated in brackets. Asterisks mean significant differences in the learning performance (*p* < 0.001). Nonanal was used as novel odor during the testing phase (NO).

**FIGURE 4:**
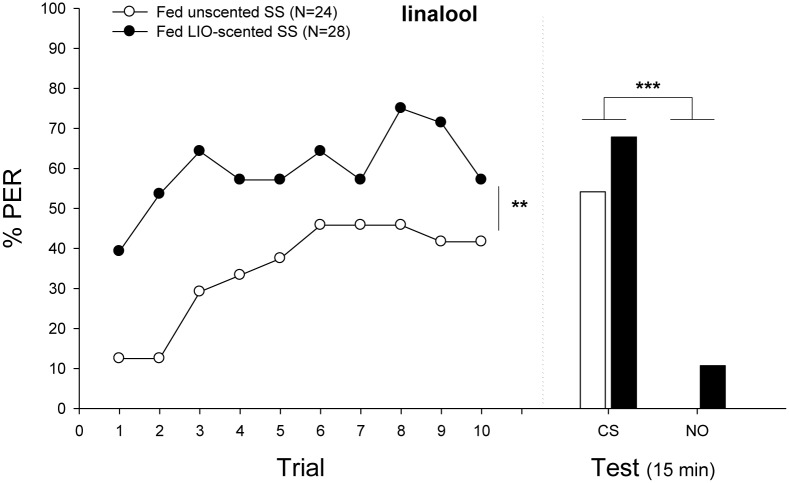
Experiment 3: Effects of scented food in bumblebees classical conditioning. Percentage of bees that extended the proboscis as response to the odorant (% PER) during the ten trails in which the conditioned odor was paired with sucrose reward (training, **left panel**) and bees that responded during a testing period 15 min after training (test, **right panel**). Bumblebees were fed either sucrose solution (SS) scented with linalool (CS, filled circles) or unscented sucrose solution (emptied circles) before classical conditioning. In the training phase, learning performance of exposed bumblebees increased significantly (Minimal adequate model: Response ∼ Treatment + Trial + 1| ind.). In the testing phase, bees of both groups responded equally well, showing a significantly different response between odors (Minimal adequate model: Response ∼ odor + 1| ind.; unscented, emptied bars; scented, filled bars). Sample sizes are indicated in brackets. Asterisks mean significant differences in the learning performance (^∗∗^*p* < 0.01; ^∗∗∗^*p* < 0.001). Nonanal was used as NO.

**Figure 3** shows the acquisition curve of bees after an olfactory pre-exposure. The statistical analysis showed a significant effect of the interaction between treatment and trial (**Figure 4**; Minimal adequate model: Response ∼ Treatment × Trial + 1| ind; Supplementary Table S1). Then, the simple effect analyses denoted that preexposed bees initially exhibited decreased learning compared with unexposed bees (Trial 1 vs. Trial 2: Control, *Z*-value = 3.768, *p* < 0.05; Preexposed, *Z*-value = 1.149, *p* = 0.9998). Throughout trials, bees of both groups achieved a high level of response, showing no significant differences in the retention performance (Minimal adequate model: Response ∼ odor + 1| ind., *p* < 0.001).

#### Experiment 3: Prestimulation With Scented Food

**Figure 4** shows the acquisition and retention performances of individuals exposed or not to LIO. When odor exposure was paired with sucrose reinforcement prior to conditioning, bumblebees exhibited a higher performance throughout trials (Minimal adequate model: Response ∼ Treatment + Trial + 1| ind.). Preexposed individuals showed a high initial level of response (39% of bees that extended the proboscis during the first presentation of the odor), reaching a level of 57% at the tenth trial. On the contrary, the acquisition curve of unexposed bees was similar to the one obtained when bees were conditioned to LIO or PHE (see section “Results”). No such asymmetry was found in the retention performances of both groups. Individuals learned equally, showing a significantly different response between odors but not between treatments (Minimal adequate model: Response ∼ odor + 1| ind., *p* < 0.001).

## Discussion

Our study demonstrates that the South American native bumblebees *B. atratus* possess clear abilities to associate an *a priori* neutral stimulus with reinforcement. We showed that workers of this species, in an olfactory classical PER conditioning protocol can learn a pure odor when it was presented in paired association with a sugar reward, regardless of the odor identity, in this case, LIO or PHE. In addition, when we analyzed the influence of the previous olfactory experiences, bees showed a decrement in learning performance resulting from the non-reinforced pre-exposure in the rearing environment to the to-be-conditioned odor. Nevertheless, when a scented food was administered, workers improved their learning performance during PER conditioning to the known odor. The variability in the learning acquisition curves observed in the different control series could be due to a seasonal, as it was observed in honey bees (Lehmann et al., 2011), or to a colony effect. To avoid the first situation we performed control groups for each experimental series corresponding to the different experimental series. To discard the latter situation, we ensured that multiple colonies were used in each experiment and both treatments were assigned to the allocated colonies. Moreover, *B. atratus* individuals were able to perceive and learn the odor used as novel odor during the all experimental series, Nonanal, ruling out a possible asymmetry odor perception.

The ability of bumblebees to associate a specific odor with a sucrose solution constitutes the basis for learning that certain flowers provide nectar rewards and, consequently, for identifying the most profitable food resources. In this respect, we showed that *B. atratus* workers can establish this association, reaching a level of more than 50% correct responses after ten training trials. Our results are consistent with those reported in *B. terrestris* by Sommerlandt et al. (2014) (ca. 60%), but not with Laloi and Pham-Delègue (2004) (ca. 30%). Furthermore, our results differ from Riveros and Gronenberg (2009) whose study was performed on *B. occidentalis* (ca. 85%). This variable learning performance in bumblebees could be due to the different methodologies carried out, as a different intertrial interval (ITI) during conditioning or hours spent in the incubator (Toda et al., 2009).

When we set out to evaluate the influence of previous olfactory experiences in the learning performance of bumblebees workers *B. atratus*, we found dissimilar effects depending on the presence of the scent either as a volatile in the rearing environment (without pairing with the unconditioned stimulus, US) or diluted in the food (associated with the reward). Our results showed that olfactory exposure in the environment 1.5 h prior to conditioning, delayed the establishment of a predictive relationship between the exposed odorant and the reward during a later PER conditioning procedure, as a consequence of a latent inhibition effect (as in honey bees, Chandra et al., 2000; Fernández et al., 2009). This, defined by Lubow (1973), is a phenomenon in which the first-learning information interferes with memory for the second-learning one. Thus, it makes that subjects that have been preexposed to a CS without reinforcement delay the conditioned response when the CS is paired with the US. This is the first report about the presence of latent inhibition in bumblebees. In contrast with our results, other studies found the occurrence of sensory priming in bees, a non-associative phenomenon, after an odor pre-exposure (Molet et al., 2009; Roselino and Hrncir, 2012). Roselino and Hrncir (2012), working with stingless bee foragers *Melipona scutellaris*, found that repeated, albeit unrewarded, presentation of an odor significantly influenced the subsequent food choice of foragers, biased toward the preexposed odor. Likewise, Molet et al. (2009) showed that the presence of a floral scent in the nest environment in the absence of a reward is itself sufficient to bias the landing preference of *B. terrestris*, even if the exposure time is short, suggesting that bees either had learned the volatile scent or had been sensory-primed by perceiving it. Since these authors did not prevent the possible contact with honeypots inside the nest, the association of the odor and the honey reward could not be ruled out.

On the other hand, when odor exposure was paired with sucrose reinforcement prior to conditioning (20.5 h beforehand), bumblebees increased their responses to the CS during trials, due to the fact that food odor acts as a previous stimulus (current study). Our results are consistent with those reported by McAulay et al. (2015), who demonstrated that contacts with scented food inside the *B. impatiens* nest, increased the likelihood a bee would respond to the scent. Even more, individuals that failed to contact a honeypot containing the scented sucrose solution exhibited no response to the known scent. On the contrary, as we mentioned above, Molet et al. (2009), in *B. terrestris*, showed that the pre-exposure to an unrewarded odor is sufficient to promote preferential landings on artificial scented flowers. The fact that different bumblebee species were involved and the odors used (anise, peppermint vs. 2-phenylethanol, methyl salicylate: which could differ in their salience) may account for the discrepancies between the studies above mentioned. Additionally, while Molet et al. (2009) tested short-term memory (within an hour), McAulay et al. (2015) evaluated long-term memory (three and 6 days). Such dissimilar time span could trigger neural changes which become consolidated or not according to the presence/absence of association of the scent with a reward.

Finally, an alternative explanation for the improved learning performance of bumblebees preexposed to scented food would involve sensory pseudoconditioning. This phenomenon could be ruled out since the control group (fed with unscented sucrose solution prior to training) did not show such positive effect in the acquisition, suggesting that the improvement found would be the consequence of the previous odor-reward association (Mackintosh, 1994), instead of an alternative effect.

Concerning social learning, in both stingless bees and honey bees, appetitive learning (scent associated with a gustatory reward; Takeda, 1961; Bitterman et al., 1983; Menzel, 1999) via trophallactic food exchanges with successful foragers influences the foraging decisions of individuals naïve to food sources (*stingless bees:* Jarau, 2009; Mc Cabe and Farina, 2009; Mc Cabe et al., 2015; *honey bees:* Farina et al., 2005, 2007; Farina and Grüter, 2009; Balbuena et al., 2012a). In contrast, bumblebee foragers do not perform trophallaxis and cannot communicate spatial information about rewarding food sources, but they can provide odor information from rewarding flower species to their nestmates. In these insects, the crop unloading is done directly into the honeypots by the foraging bee (Dornhaus and Chittka, 2005). These honeypots are the source of the olfactory information stored inside of the colony because a bumblebee probes the nectar contained in them and then goes out to forage (Dornhaus and Chittka, 2005).

Despite the fact that our results do not demonstrate that bumblebees *B. atratus* are capable of social learning, like numerous other social insects (*honey bees:* Farina et al., 2005, 2007; Balbuena et al., 2012a,b; *bumblebees of other species:* Dornhaus and Chittka, 1999; Molet et al., 2009; McAulay et al., 2015), the present study is the first step to understand the mechanisms involved in the recruitment and communication capacity of this particular bumblebees species. Future research may focus on learning associations of scents and food stored in honeypots within the bumblebee nest, where the information transfer takes place, to evaluate its social learning capacity. Such studies on the associative conditioning of floral odors and a sucrose reward could be useful as a tool to influence the foraging behavior of bumblebee workers, opening the possibility to improve the nest management during the pollination services. Furthermore, it is a matter of relevance bearing in mind the fact that *B. atratus* is increasingly reared as an alternative native species and the potential risk of invasion by exotic bumblebees.

## Author Contributions

FP, ME, and WF: conceived and designed the experiments and wrote the article. FP and ME: performed the experiments and analyzed the data. WF: contributed reagents/materials/analysis tools.

## Conflict of Interest Statement

The authors declare that the research was conducted in the absence of any commercial or financial relationships that could be construed as a potential conflict of interest.
