# Supplementary material for: Odor Learning and Its Experience-Dependent Modulation in the South American Native Bumblebee Bombus atratus (Hymenoptera: Apidae)
Source: Front Psychol. 2018 Apr 27;9:603. doi: 10.3389/fpsyg.2018.00603 (PMC5934905; doi:10.3389/fpsyg.2018.00603)
Supplement: Supplementary file 1 [file Table_1.pdf]

Supplementary Material

Odor Learning and its Experience-dependent Modulation in the South American Native Bumblebee *Bombus atratus* (Hymenoptera: Apidae)

Florencia Palottini, María Cecilia Estravis Barcala and Walter Marcelo Farina*

*Correspondence: walter@fbmc.fcen.uba.ar

Table S1. Set of variables considered in the generalized linear mixed effects models explaining bumblebees' responses in the different experiments. The p-values are extracted from the comparison between nested models (by using the anova function).

Experiment	Phase	Variable	Chi sq	P-value
1- Olfactory classical conditioning:<br>A) Linalool as conditioned stimulus	Training	Treatment	31.4	**$2.1e^{-08}$**
		Trial	38.598	**$4.154e^{-09}$**
	Test	Treatment	14.271	**$1.583e^{-04}$**
		Odor	28.625	**$8.783e^{-08}$**
1- Olfactory classical conditioning<br>B) Phenylacetaldehyde as conditioned stimulus	Training	Treatment	22.81	**$1.789e^{-06}$**
		Trial	19.954	**$4.647e^{-05}$**
	Test	Odor	26.513	**$2.618e^{-07}$**
2- Volatile preexposure	Training	Treatment x Trial	28.81	**$6.977\ e^{-04}$**
	Test	Treatment	0.3282	0.5667
		Odor	40.864	**$1.632e^{-10}$**
3- Prestimulation with scented food	Training	Treatment	4.1719	**$4.11e^{-02}$**
		Trial	11.664	**$2.932e^{-03}$**
	Test	Treatment	2.6588	0.103
		Odor	40.636	**$1.834e^{-10}$**
4- Olfactory classical conditioning (Figure S1)	Training	Trial	35.104	**$2.384e^{-08}$**
	Test	Odor	29.797	**$4.797e^{-08}$**

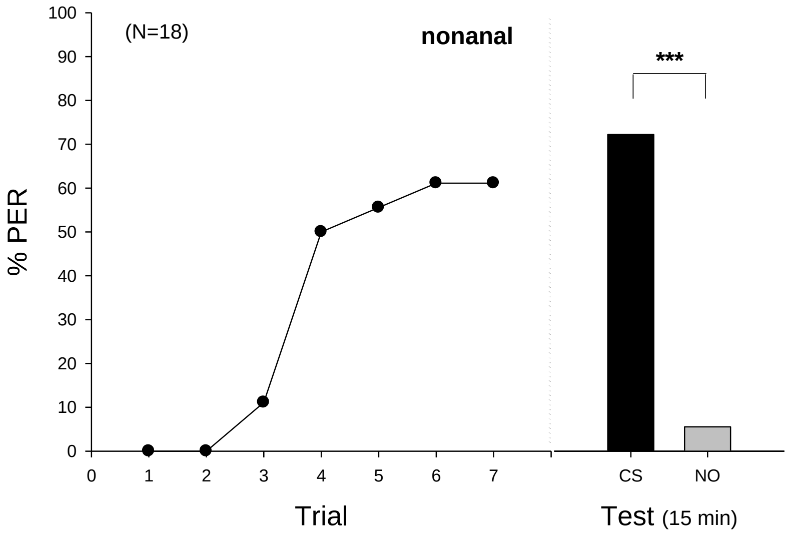

FIGURE S1. Olfactory classical conditioning of proboscis extension in bumblebees. Percentage of bees that extended the proboscis as response to the odorant (% PER) during seven trails (training, ***left panel***) and bees that responded during a testing period 15 min after training (test, ***right panel***). Bees trained with nonanal as the conditioned stimulus (CS) and linalool as novel odor (NO), showed a high performance (*ca.* 60%) during training, excluding a possible asymmetric odor perception. Sample sizes are indicated in brackets. Asterisks mean significant differences in the learning performance ($p < 0.001$).
